# Navigating with peripheral field loss in a museum: learning impairments due to environmental complexity

**DOI:** 10.1186/s41235-019-0189-9

**Published:** 2019-10-22

**Authors:** Erica M. Barhorst-Cates, Kristina M. Rand, Sarah H. Creem-Regehr

**Affiliations:** University of Utah, Salt Lake City, UT USA

**Keywords:** Spatial learning, Restricted peripheral field, Complex environment

## Abstract

**Background:**

Previous research has found that spatial learning while navigating in novel spaces is impaired with extreme restricted peripheral field of view (FOV) (remaining FOV of 4°, but not of 10°) in an indoor environment with long hallways and mostly orthogonal turns. Here we tested effects of restricted peripheral field on a similar real-world spatial learning task in an art museum, a more challenging environment for navigation because of valuable obstacles and unpredictable paths, in which participants were guided along paths through the museum and learned the locations of pieces of art. At the end of each path, participants pointed to the remembered landmarks. Throughout the spatial learning task, participants completed a concurrent auditory reaction time task to measure cognitive load.

**Results:**

Unlike the previous study in a typical hallway environment, spatial learning was impaired with a simulated 10° FOV compared to a wider 60° FOV, as indicated by greater average pointing error with restricted FOV. Reaction time to the secondary task also revealed slower responses, suggesting increased attentional demands.

**Conclusions:**

We suggest that the presence of a spatial learning deficit in the current experiment with this level of FOV restriction is due to the complex and unpredictable paths traveled in the museum environment. Our results also convey the importance of the study of low-vision spatial cognition in irregularly structured environments that are representative of many real-world settings, which may increase the difficulty of spatial learning while navigating.

## Significance

Navigating in unfamiliar spaces is often challenging for normally sighted people and becomes even more demanding for those with severe visual impairment. Limitations in the ability to learn the spatial layout of new spaces have potentially negative consequences of reducing independence and increasing social isolation of those with low vision. While basic and applied research has studied aspects of low vision navigation such as mobility, distance perception, and spatial learning, much of this work has been studied in simple environments that are not always representative of the complexity of everyday life. Our current study examines spatial learning in a real-world art museum under severely restricted peripheral field of view (FOV). We consider how spatial learning is affected by cluttered and complex paths that are not typical of laboratory settings. This approach is important for understanding the optimal design of navigational assistive devices for the visually impaired that are adaptable to the varying environments in which people navigate on a daily basis.

## Introduction

With a growing older adult population and the subsequent rise of clinically defined low vision (https://nei.nih.gov/eyedata/), it is important to understand ways to improve or at least maintain ability for visually impaired individuals to complete every-day tasks, such as navigation through an indoor environment. Low vision describes an uncorrectable visual impairment that does not result in complete blindness, allowing some residual vision to be used for daily functions. Spatial learning of novel environments is a challenge with low vision, one that may become even more difficult with complex and less predictable environments. Prior work from our laboratory has shown that spatial learning during navigation is impaired with both simulated severely degraded acuity and contrast sensitivity (Rand, Creem-Regehr, & Thompson, 2015) and severely restricted peripheral FOV (Barhorst-Cates, Rand, & Creem-Regehr, 2016) in a real-world environment, and has attributed the deficit in learning partially to the attentional demands of monitoring to ensure safe mobility (Barhorst-Cates et al., 2016; Rand et al., 2015). Much of the prior work on low-vision spatial perception and navigation has used large, highly structured indoor hallways (Barhorst-Cates et al., 2016; Barhorst-Cates, Rand, & Creem-Regehr, 2017; Rand et al., 2015) or single-room environments (Fortenbaugh, Hicks, Hao, & Turano, 2007; Fortenbaugh, Hicks, & Turano, 2008; Legge, Gage, Baek, & Bochsler, 2016; Legge, Granquist, Baek, & Gage, 2016; Yamamoto & Philbeck, 2013). However, everyday navigation often occurs outside the context of straightforward hallways or rooms and it is unknown how low vision affects spatial learning in more irregular spatial contexts. Our goal was to test the influence of restricted peripheral field on spatial learning in more complex environments, specifically with regards to the greater complexity of navigation paths. We predicted greater decrements in spatial learning than have been seen previously in the relatively simple topological structure and sparsely populated environments more typical of laboratory settings.

Peripheral field loss increases the challenges of navigation in complex indoor spaces because of its impact on both visual search for landmarks and the integration of component visual features of the environment into a spatial structure (Fortenbaugh et al., 2008). Viewers are unable to view the entire scene at once and must incorporate multiple eye or head movements to gather information from both central and peripheral sources (Yamamoto & Philbeck, 2013). Barhorst-Cates et al. (2016) showed that these challenges of navigation with peripheral field loss make the task of spatial learning more cognitively demanding at severe levels of restriction (when remaining FOV is 10°). However, participants were shown to learn the locations of novel targets at the same level of accuracy as with normal vision until faced with extreme (4° FOV) restrictions. It is possible that spatial learning while navigating with severe 10° FOV restriction was facilitated by predictable environments in this previous experiment. That is, the participants in Barhorst-Cates et al. (2016) performed a real-world indoor spatial learning task in a highly structured building with paths down long narrow hallways with a few 90° turns, and were likely able to use multiple sources of information to aid with memory that are not available in environments with more complex and cluttered layouts.

In normal viewing contexts, several studies support the claim that spatial knowledge is facilitated by environmental regularity, defined as straightness of a path and orthogonality of turns on a route. People in the USA perform better on multi-turn navigation tasks that contain orthogonal rather than oblique turns (Sadalla & Montello, 1989) and even tend to remember oblique turns as orthogonal in map drawing tasks (Byrne, 1979; Lynch, 1960; Moar & Bower, 1983; Tversky, 1981) and turn reproduction tasks (Sadalla & Montello, 1989). People also have more accurate knowledge of the environment when tested for their accuracy in pointing to landmarks in familiar places at orthogonal rather than oblique turns in a city (Montello, 1991). Additionally, people prefer shorter, direct routes with few turns (Dalton, 2003; Golledge, 1995) and perform better on navigation tasks that require fewer turns (He, McNamara, Bodenheimer, & Klippel, 2019). For example, in a virtual navigation task, participants were more accurate on a pointing task in environments that were simple with fewer turns compared to environments that were more complex (i.e., misaligned with the viewpoint of the observer) with more necessary turns (He et al., 2018). The authors argue that accuracy decreases with greater numbers of turns because of accumulated error during path integration, the updating of current self-location in an environment (Kelly, McNamara, Bodenheimer, Carr, & Rieser, 2008; Rieser & Rider, 1991). This path integration error can be reduced when the environment has sufficiently complex, stable, and informative visual information, which could encourage a shift from body-based reliance to visual, or “landmark” reliance (Zhao & Warren, 2015a, 2015b). Taken together, in normal viewing conditions people seem to prefer and perform best on navigation tasks through structured, predictable environments with few orthogonal turns, during which they can rely on both visual and body-based information.

Little is known, however, about how spatial learning while navigating with *viewing impairment* is influenced by the complexity or predictability of paths taken through a novel environment. In addition to our prior work examining peripheral field loss in structured environmental navigation contexts (Barhorst-Cates et al., 2016), there has been some work examining the effect of peripheral field loss on spatial learning in open indoor environments (Legge, Gage, et al., 2016; Yamamoto & Philbeck, 2013). In a study by Legge, Gage, et al. (2016), participants with various types of simulated low vision, including restricted FOV of 8°, completed judgments of room size and a spatial updating task in which they had to return to a home starting position after traveling a path within an environment. Room size judgments were impaired in the narrow FOV condition compared to normal vision, but spatial updating was not impaired with FOV restriction in either walking or wheelchair conditions. As such, a wide FOV does not seem necessary to complete simple spatial updating tasks, but it does affect other properties of environmental learning such as perceived scale of a room (Legge, Granquist, et al., 2016) and distance perception (Fortenbaugh et al., 2007, 2008).

There are several factors that likely contribute to the functional complexity of an indoor environment, including structural components of the environment itself (Carlson, Hölscher, Shipley, & Dalton, 2010). Museums are spaces that are often designed to be fairly unstructured in potential paths with purposeful dead-ends so that visitors can choose different routes through exhibits, interacting with the space in a dynamic way (Peponis, Dalton, Wineman, & Dalton, 2004). At any given location in a museum (at least the museum used in the current study), there are many choices for possible routes that are typically visually connected. Peripheral field restriction inherently alters the ease of viewing connected spaces in the environment, which makes the study of the interaction of environmental structure and visual deficit even more relevant. Carlson et al. (2010) argue that complexity is defined as the interaction of these structural components of a space with the navigator’s strategies/abilities and mental representation of the environment. These strategies and representations likely change in those navigating with peripheral field loss, as they are challenged with additional demands both in integrating multiple views (Yamamoto & Philbeck, 2013) and maintaining safe mobility during locomotion (Barhorst-Cates et al., 2016). In addition to the added challenges from layout complexity in any environment, the museum environment presents unique challenges related to obstacle avoidance and perceived risk. The potential for collisions with valuable art is likely to further increase attentional demands and impact spatial learning. Given our previous work demonstrating the role of mobility monitoring during navigation with low vision even in fairly obstacle-free environments, this higher mobility-risk context should also contribute to decrements in spatial learning.

Walking through a space, rather than stationary viewing, adds complexity to the task of navigating that can both facilitate and impair learning through different mechanisms. Movement facilitates spatial learning by allowing for automatic spatial updating of position and active encoding of spatial relations (Rieser, 1989). In our prior work on peripheral field loss in navigation, we argue that the information provided by active locomotion through the hallways could be used for spatial learning of target locations and partially compensated for the loss of visual information. This is a potential explanation for the intact performance seen at a severely restricted FOV of 10° (Barhorst-Cates et al., 2016). However, active walking also comes at a cost when navigating with impaired vision by requiring additional attentional resources - a process termed “mobility monitoring” (Rand et al., 2015) - to maintain safety while walking (to avoid obstacles, maintain balance, etc.). Taken together, it is feasible to suggest that errors in perception of environmental scale combined with increased attentional demands may impair spatial learning ability. Attentional demands may arise due to both spatial integration of features across views and mobility monitoring with reduced FOV, especially in environments that are unpredictable in nature. Here, we incorporated paths with short segments and many turns in an attempt to increase the mobility monitoring demands and reduce the facilitative effects of the long hallways that may have aided spatial learning in Barhorst-Cates et al. (2016). To our knowledge there is no study that has examined effects of restricted peripheral field on spatial learning in complex, indoor environments in this way.

The aim of the current study was to measure spatial learning performance with restricted FOV in a real-world, complex, indoor environment. We predicted that participants would make larger spatial learning errors when they learned paths with a simulated severe restriction of 10° FOV compared to learning paths with a mild restriction of 60° FOV. We also expected participants to have greater cognitive load while navigating with severe compared to mild restrictions. We predicted the spatial learning results to differ from those of Barhorst-Cates et al. (2016) with the same FOV restriction that was conducted in a structured environment, arguing for the added difficulty of navigating in complex environments with FOV loss.

## Method

### Participants

Participants (*n* = 32, 23 women) were recruited from the University of Utah psychology department participant pool and received partial course credit for completing the study. The average age was 23.8 (SD = 8.6) with a range of 18–60 years. All participants gave written informed consent with procedures approved by the University of Utah Institutional Review Board. All participants had self-reported normal or corrected-to-normal vision and walked without impairment.

### Materials

Participants wore the same goggles as reported by Barhorst-Cates et al. (2016), which restricted the peripheral FOV of the dominant eye, resulting in either severe (10°, referred to as narrow) or mild (60°, referred to as wide) remaining FOV. Fixed, peripheral-field occluders such as these cause problems with binocular viewing, because the region in which stereo fusion is possible is limited. As a result, we used monocular viewing with the dominant eye, and completely covered the non-dominant eye. The goggles were welding goggles with black cardstock paper covering the non-dominant eye and with the appropriate sized hole cut out on the side of the dominant eye (3 mm diameter for the narrow FOV and 42 mm diameter for the wide FOV). They provide an average FOV of 11.14° (narrow FOV) and 67.9° (wide FOV) because of variability in head size and placement on the head (Barhorst-Cates et al., 2016). Each participant wore both sets of goggles in the experiment.

### Procedure

Researchers met participants in the lobby of the Utah Museum of Fine Arts (UMFA) and participants filled out written consent and demographics forms. Then researchers explained the degree-quadrant pointing task (Philbeck, Sargent, Arthur, & Dopkins, 2008). Participants were instructed to stand in the center of a space and imagine four quadrants around them, front-left, front-right, back-left, and back-right. Each quadrant is divided into a range from 0 to 90°. For the front two quadrants, 0° is straight ahead and 90° is straight to the sides. For the back two quadrants, 90° is straight behind and 0° is straight to the sides. After the experimenter drew an overview of this degree-quadrant system, participants were asked to stand up and practice. The experimenter placed a pen at various points around the participant and asked him or her to point to the pen using the quadrant and degree system. Each participant practiced pointing to the pen at least once in each quadrant and practice was terminated once participants understood. Next, participants completed a dominant eye test to determine which goggles they would be using. Then experimenters explained the secondary auditory reaction time (RT) task. Participants were instructed to listen through headphones to a series of randomized beeps that occur every 1–6 s. Upon hearing a beep, participants were instructed to click a wireless mouse once as quickly as possible. Responses were recorded on a laptop carried by a second experimenter throughout the experiment.

Next, participants were led into the museum to begin practice trials. Participants practiced walking while wearing each type of restricted goggles and practiced responding to beeps while walking with the goggles. Throughout all practice and experimental paths, participants held onto the experimenter’s arm to avoid collision with the art pieces. Participants completed two practice paths, one in each set of goggles, in which they were guided along a path with various turns by the experimenter and learned the location of two pieces of art. At the end of the path, participants pointed to the remembered location of the targets using the degree-quadrant system, as if they could point directly through the walls. Upon completion of the practice paths, participants were given the chance to ask any and all questions before being led to the start of the first experimental path. Each participant completed four experimental paths, which did not overlap with the practice paths, one on the first floor of the museum and three on the second floor. Vision condition alternated between paths and was counterbalanced between participants, such that half of participants completed the paths in a wide-narrow-wide-narrow order and the other half of participants completed the paths in a narrow-wide-narrow-wide order.

**Fig. 1:**
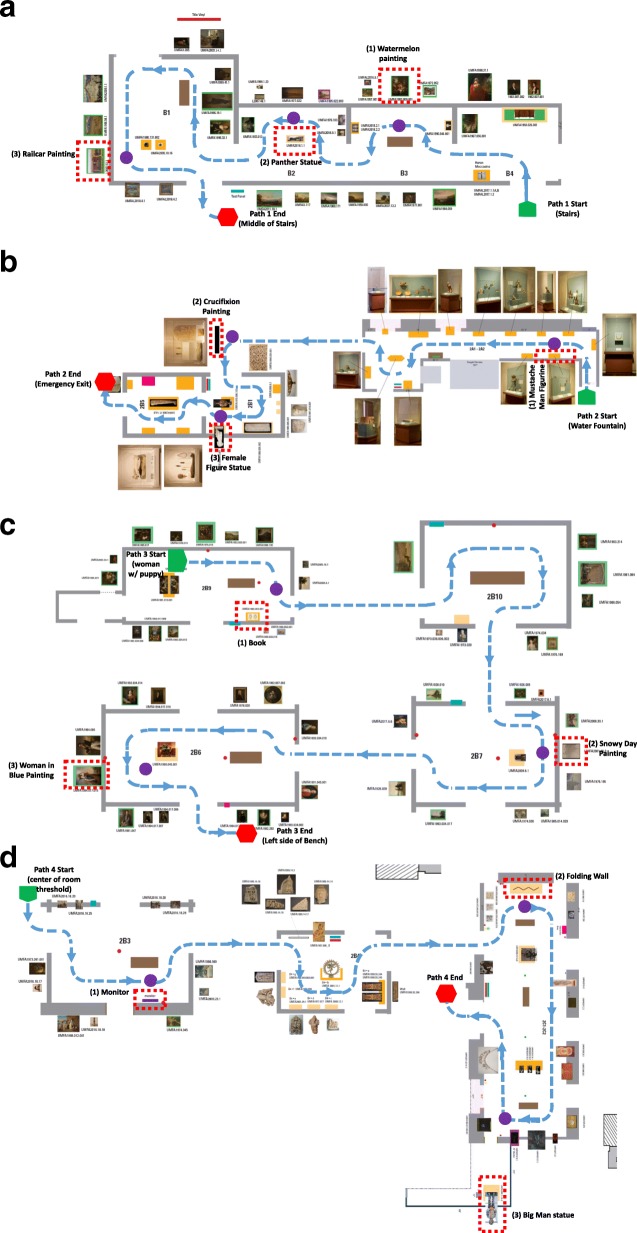
**a** Path 1 from the Utah Museum of Fine Arts. Participants were guided along and stopped at each of the three target art pieces - the watermelon painting, panther statue, and railcar painting. They pointed to each target from the end of path 1 during recall. Each path was similarly complex with many turns into alcoves. Rather than turning sharply right or left, the paths necessitated smoother curved turns. At any point along the path, the possible choices for routes varied (i.e., to go straight, turn right into an alcove, go straight then turn right and circle back around an alcove, etc.), which made the paths less predictable and structured in nature. **b** Path 2 from the Utah Museum of Fine Arts. **c** Path 3 from the Utah Museum of Fine Arts. **d** Path 4 from the Utah Museum of Fine Arts

To quantify path complexity in this environment, we used our pre-determined paths and calculated the ratio of turns/total path distance. The direction of each target relative to the participant as it was encountered on the path is in parentheses following the target name. Path 1 had 15 turns and included three targets, a painting of a watermelon (right), a statue of a panther (left), and a painting of a railcar (right; see Fig. 1a). The turns/distance ratio was 0.25. Path 2 had 16 turns and included three targets: a figurine of a man with a mustache (left), a painting of the crucifixion (right), and a statue of a female figure (left; see Fig. 1b). The turns/distance ratio was 0.25. Path 3 had 18 turns and included three targets: an ancient book (right), a painting of a snowy day (left), and a painting of a woman in blue (right; see Fig. 1c). The turns/distance ratio was 0.28. Finally, path 4 had 16 turns and included three targets: an electronic television monitor playing an art video (right), a large folding wall (left), and a statue of a large man (right; see Fig. 1d). The turns/distance ratio was 0.22. No path intersected with any other path and paths had an average length of 63.4 m, taking about 3 min to walk. As a comparison, the paths used in Barhorst-Cates et al. (2016) with sparse hallways had four or five turns with turns/distance ratios of 0.03, 0.03, 0.05, and 0.04. An independent samples *t* test confirmed that the paths in the UMFA had a significantly higher ratio of turns for total distance (mean (M) = 0.25, SD = 0.03, *t* = 16.16, *p* < .001) compared to the paths in our prior study (M = 0.04, SD = 0.01). At the end of each path, participants pointed to the location of the remembered targets in a random order as prompted by the experimenter. They then completed 1 min of the listening task alone to establish baseline RT performance before being led to the beginning of the next path. Participants were asked to close their eyes to ensure that they did not view the upcoming path in advance. Experimenters then guided the participant to the start of the next path with the participant’s eyes closed. After participants completed all 4 paths, they were led back into the lobby of the museum and filled out the Santa Barbara Sense of Direction scale (SBSOD; Hegarty, Richardson, Montello, Lovelace, & Subbiah, 2002). They were then debriefed, thanked, and dismissed.

## Results

**Fig. 2:**
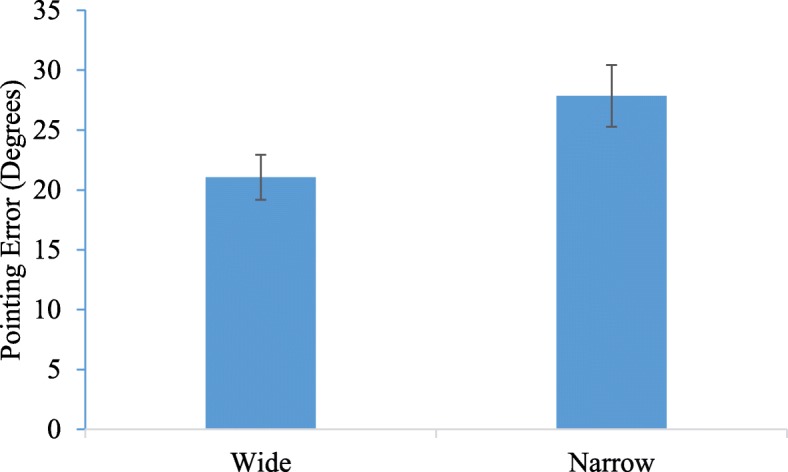
Comparison of spatial learning error in wide and narrow field of view (FOV) conditions in the Utah Museum of Fine Arts

Pointing error (unsigned) was calculated as the absolute value of the difference between the participant’s response pointing direction and the correct pointing direction. An average pointing error was calculated for each individual for each vision condition (two paths each in wide and narrow FOV with three targets per path resulting in six pointing responses constituting the average per vision condition). Two outliers with average pointing error greater than 3 SD above the mean were identified (one for the wide condition, one for the narrow condition). These participants were removed from the following analyses. We ran a 2 (Vision Condition: Narrow vs. Wide) × 2 (Vision Order: Narrow First vs. Wide First) repeated measures analysis of variance (ANOVA) with vision condition manipulated within subjects and vision order manipulated between subjects and pointing error as the outcome variable. We found a significant main effect of vision condition *F* (1,28) = 4.23, *p* < .05, *η*_*p*_^2^ = 0.13 with significantly higher pointing error in the narrow FOV condition (M = 27.1, SE = 2.50) compared to the wide FOV condition (M = 20.73, SE = 1.82) (See Fig. 2). There was no main effect of vision order (*p* > .9) and no vision condition X vision order interaction (*p* > .6). The pointing error results are in contrast to the findings of Barhorst-Cates et al. (2016) in which the same FOV manipulation and paradigm were used but in a sparsely populated building with paths along long hallways with few degree turns, and which did not find a detriment in pointing accuracy when comparing 10° narrow FOV (M = 20.75) to wide FOV (M = 19.74).

We ran the same 2 (Vision Condition: Narrow vs. Wide) × 2 (Vision Order: Narrow First vs. Wide First) repeated measures ANOVA with average RT as the outcome variable. A slower RT implies greater cognitive load (Verwey & Veltman, 1996). We observed a significant main effect of vision condition, *F* (1,28) = 7.78, *p* < .01, *η*_*p*_^2^ = 0.22, such that participants were significantly slower to respond in the narrow FOV condition (M = 0.61, SE = 0.02) compared to the wide FOV condition (M = 0.59, SE = 0.02). There was no main effect of vision order (*p* > .09) but there was a significant vision condition X vision order interaction *F* (1,28) = 8.54, *p* < .01, *η*_*p*_^2^ = 0.23. Post-hoc paired *t* test demonstrated that the effect was driven by those who completed the narrow FOV condition first. Those who completed the narrow condition first had faster responses (*t* = − 5.11, *p* < .001) in wide (M = 0.61, SE = 0.03) versus narrow (M = 0.65, SE = 0.03) FOV. Those who completed the wide condition first did not exhibit a significant difference (*t* = 0.08, *p* > .9) between wide (M = 0.57, SE = 0.03) and narrow (M = 0.57, SE = 0.03) RT, suggesting experience first with the wide FOV may have reduced the impact on cognitive load with the subsequent narrow FOV.

Score on the SBOD scale was significantly negatively correlated with narrow FOV pointing error (*r* = −.56, *p* < .01) but was not correlated with wide FOV pointing error (*r* = −.23, *p* > .2). This suggests that a person’s self-reported sense of direction predicts their pointing accuracy in conditions of severe visual restriction, but it may not be indicative of performance when vision is less restricted.

## Discussion

Our aim was to use a naturalistic museum setting to test the influence of severe peripheral field restriction on spatial learning while navigating. This approach stands in contrast to much of the previous work examining low-vision mobility and navigation, which has used regular hallway environments that are not always representative of the complexity of real-world navigational challenges. Previous work in structured hallways suggests that while navigating through a novel space by people with visual impairment is cognitively demanding, spatial learning may be supplemented by the additional body-based cues associated with walking and turning in regular and predictable ways (Barhorst-Cates et al., 2016). In the current study, participants completed a spatial learning task in an art museum wearing goggles that simulated severe peripheral field restriction (10° FOV) compared to completing the task with mild FOV restriction (60° FOV). We found support for our predictions that spatial memory errors (revealed by pointing to remembered landmarks) and cognitive load would increase in the narrow compared to the wide FOV condition. We argue that the presence of a spatial learning deficit in the current experiment with this level of FOV restriction, unlike Barhorst-Cates et al. (2016), is influenced by the context of the environment and complexity of the required navigational paths.

The increase in spatial memory error seen in the narrow versus wide FOV condition in the museum context may have been due to a combination of visual, motor, and attentional influences on learning, related to the museum path structure. The visual components of navigating with field loss make the task more visually demanding, recruiting more head/eye movements to integrate reduced views of the scene. Additionally, the information provided by physical movement may be less helpful to rely on than in simpler environments, which would have a greater detrimental effect when visual information is reduced. Finally, attentional demands on a navigator are increased both because of the need to integrate segments of a route with less distinct sub-pieces, and because of increased need for monitoring one’s own mobility with low vision. We discuss each of these factors in the following sections.

First, spatial learning with FOV restricted to 10° may be significantly impaired in the museum space because the environment cannot be viewed all at once. In more typically studied hallway or stationary-viewing environments, viewing with peripheral field loss requires increased head rotation and integration of multiple views, which results in worse performance (Barhorst-Cates et al., 2016; Fortenbaugh et al., 2007; Yamamoto & Philbeck, 2013). We suggest that navigation in a museum further increases these visual demands because of the open nature of the environment, affecting the overall intelligibility, or mutual visibility (Hillier, 2006), of the space. In other research in complex, open environments, Hölscher, Brösamle, and Vrachliotis (2012) found that spaces with insufficient access to visual information across the space and unexpected navigational features, such as dead-ends or unexpected staircases, negatively impaired navigation ability. In a library wayfinding task, Li and Klippel (2016) also show that visual accessibility is a vital component of the “environmental legibility” of a space, arguing that visual information can facilitate spatial knowledge even in complex spaces. While the current study focused specifically on path-complexity spatial learning with restricted FOV, other features of the complex museum environment such as intelligibility likely also increase navigation demands in low vision. Quantifiable measures of visibility should be incorporated into future research and in the design of navigation aids for the visually impaired. It may also be that distances traveled were perceived incorrectly or inconsistently along different path segments due to the reduced FOV effects on perceived self-motion, as has been seen with reduced acuity and contrast sensitivity (Rand, Barhorst-Cates, Kiris, Thompson, & Creem-Regehr, 2018), contributing to overall error in memory for spatial layout. These vision-related components of navigation were not the primary aim of the current study, but should be tested in future research by analyzing visibility in test buildings, measuring head/eye movements, and testing distance perception.

In order to view art pieces in a museum, people take circuitous routes that incorporate many turns and lead viewers along short individual paths into purposeful dead-ends (Peponis et al., 2004), increasing demands for learning and memory. In this study we attempted to mimic natural museum behavior by including many turns that moved in and out of alcoves and around obstacles such as benches and art pieces in the center of the room. The paths in this experiment had many turns that were not all orthogonal, with several possible directions to move at any given time. This increased task difficulty by (1) providing less predictable route choices and (2) reducing the distinctiveness of decision points along the route. This may affect both the encoding and recall components of the task due to well-known decrements in cognitive map formation in complex environments (Byrne, 1979; Lynch, 1960; Moar & Bower, 1983; Tversky, 1981) as well as consequences arising from self-motion information through complex environments. While encoding the natural museum routes, a navigator may not be able to make accurate predictions of upcoming turns while learning, and turns are rarely orthogonal right or left turns. If a navigator used a strategy of remembering key turns along a route, having more turns to remember would inherently create more conflict in memory. Those non-orthogonal turns that are remembered might also be distorted in their representation in memory as either straight (Dalton, 2003) or orthogonal (Sadalla & Montello, 1989), as shown in prior work. In addition, while self-motion information from turns provides supplemental information for spatial learning with low vision in relatively simple environments, this information may not be as useful in the museum environment because of the need to associate target locations with one of many possible turns, as well as the increased error in path integration associated with those turns (Rieser & Rider, 1991). The body-based information from turns likely interacts with the lack of complete visual information in a way that amplifies spatial memory error. Zhao and Warren (2015b) discuss the reliability of different types of cues (visual and body-based) while navigating and argue that people shift between relying mostly on path integration (body-based cues) and mostly on visual or landmark cues depending on how reliable the visual cues are. In the case of severely restricted peripheral vision, visual cues may be less reliable or at least less detectable, which might encourage a shift to rely on body-based cues. In the museum context with many turns, this forced reliance on body-based cues may exacerbate spatial learning error due to memory conflict and accumulated path integration error. Taken together, the increased demands for learning and memory and the decreased reliability of body-based cues could explain the greater deficit in spatial memory under severely restricted viewing conditions in the museum compared to the structured hallway building.

Last, the path complexity in the environment even further increases the mobility monitoring demands that have been shown to detract from spatial learning (Rand et al., 2015). Mobility hazards could include turns, during which balance must be shifted and the body oriented in a new direction, obstacle avoidance - particularly avoidance of valuable pieces of art - and perceived changes in flooring that could affect balance or gait stability. Shorter stretches of walking could pose more threats to gait stability, as a walker would have less time to adjust and perform stable gait movements. In the museum environment, the greater number of turns that are less orthogonal in nature and the added difficulty of avoiding obstacles such as benches and centrally located art pieces all combine to create greater mobility monitoring demands compared to more structured buildings. Of note, participants were holding onto the arm of the experimenter throughout each path to minimize accidental touching of the art pieces, so in this case mobility monitoring demands were already minimized as compared to walking alone (see Rand et al., 2015). Even so, our RT dual-task measure showed increased cognitive load with the narrow compared to the wide FOV. It would be interesting in future research to examine the effects of FOV restriction on navigating with full mobility monitoring demands (i.e., without a guide) in a museum-like environment. In the current study, path complexity and target value were confounded (i.e., the museum paths all contained valuable, breakable art pieces and artifacts whereas in our prior study the targets were normal building items). It would be interesting in future research to test for the effect of valuable obstacles specifically, by testing within-subjects performance differences on similarly complex paths with non-breakable and highly valuable objects.

There are other factors that could contribute to the detriment we observed in the museum, such as building novelty or the presence of a greater number of interesting distractors. We asked participants to report their level of familiarity with the building on a scale from 1 to 7, and 87.5% of participants reported a score of 6–7, indicating little familiarity, similar to the 91.4% of participants who reported a score of 6–7 for familiarity with the building in Barhorst-Cates et al. (2016). While the potential distractors in the art museum were likely more visually appealing than those in the structured building in earlier experiments, both included the distractors that are present in a real-world university campus building in terms of people walking around, posters, advertisements, windows, and art pieces on the walls that might draw attention, etc. In an attempt to address the possible difference in the number of people present in the hallways between buildings, we purposefully scheduled the experiments at the museum to be on the days and times when large tours did not visit, so the number of people walking around in both buildings was roughly similar. Auditory distractions were also present in both cases, but markedly less so at the museum, making it the more optimal environment in that sense.

### Limitations and future directions

Our results could be explained by various influences on learning - visual, motor, attentional - that we believe all combine to create the unique, demanding task of real-world navigation. However, we cannot claim that any of these three factors alone contribute to spatial memory error in navigation with restricted peripheral field. The relative contribution of each of these factors should be systematically studied in future research by, for instance, measuring head movements to examine visual encoding behaviors or manipulating paths with more or less turns to examine the impact of turn number specifically. Admittedly, testing navigation in a complex real-world space leaves open multiple possibilities for interpretation of the factors that influence impaired spatial learning with severely restricted peripheral field. In addition to differences in environmental complexity and path complexity as compared to our previous work, the museum context introduced additional mobility challenges with increased chance of collision with valuable art. In order to tease apart effects of environmental complexity versus path complexity, future research could design more or less complex paths in both visually simple and visually complex environments using systematic space syntax analyses to assess visual complexity. Future research could also manipulate the mobility demands of navigating and the contribution of body-based cues for movement in this context by having participants locomote in a wheelchair. While there are some limitations and challenges in experimental control that are inherent in naturalistic environments, our results provide initial insights into the interaction between environmental complexity and visual restrictions in spatial learning while navigating.

Our study was motivated by the navigational challenges faced by those with visual impairment and the overarching goal of our National Institutes of Health (NIH)-funded Designing Visually Accessible Spaces project to enhance visual accessibility - the use of vision for perception of spatial layout and safe and efficient travel through spaces. A limitation is that we used simulated restricted peripheral vision to control for the amount of vision loss in this experiment. Given the challenges in accurately controlling field of view in the real world and issues with stereo fusion if viewing with two eyes, we chose to allow restricted viewing in only the dominant eye. In the clinical low-vision setting, vision loss ranges widely from person to person and often includes combinations of field loss and severely degraded acuity and contrast sensitivity at varying parts of the field-of-view, and reliance on eye movements and other strategies developed to compensate for vision loss. As such, the extent to which these simulations accurately represent people with real-world low vision is unknown. However some prior research comparing both simulated and patients with clinical low vision has identified similar effects on spatial learning (Fortenbaugh et al., 2008; Legge, Granquist, et al., 2016). Studies by Fortenbaugh and colleagues showed that both simulated and real peripheral field loss led to an underestimation of remembered distance to target locations after walking a pre-determined route in a virtual environment, and that these errors increased with decreasing FOV. However, those with real peripheral field loss also showed some differences in eye movements and fixations. Studying navigation abilities of patients with clinical low vision with field loss in variable environments is an area in much need of future research.

Beyond the low-vision motivation, our results also contribute to more broad understanding of both the role of the peripheral visual field and demands on attention for spatial learning while navigating in indoor environments. While there have been a number of studies on the influence of restricted FOV on distance perception and mobility tasks (Creem-Regehr, Willemsen, Gooch, & Thompson, 2005; Fortenbaugh et al., 2007; Pelli, 1987; Wu, Ooi, & He, 2004), there is limited work on larger-scale navigation under restricted FOV conditions. Findings in this area are especially relevant to applications using augmented reality (AR) where graphical cues can augment real spaces and potentially facilitate navigation, but the FOV of displays still remains very restricted (e.g., the state-of-the-art Microsoft Hololens has a 30 × 17° FOV). Our findings suggest that the reduced FOV may be more detrimental with complex paths or risky navigation contexts. In addition, our results supporting effects of limited attentional resources on spatial learning can be generalized beyond the specific FOV manipulation to other contexts where mobility monitoring demands are high, such as with older adults (Barhorst-Cates et al., 2017; Schellenbach, Lövdén, Verrel, Krüger, & Lindenberger, 2010) or other visually impoverished environments. Finally, we hope to be able to use the current findings identifying challenges due to path complexity to inform the development of assistive devices that could compensate for increasing attentional demands. For example, the demands of integrating multiple views could be reduced by providing auditory information about visual context outside of the field of view, or additional multisensory cues could be provided to link physical turns with salient landmarks.

## Conclusion

In conclusion, these results show that severely restricted peripheral visual field is detrimental to learning of complex paths through largely unstructured indoor environments. Furthermore, the attentional demands of navigating through environments with severe FOV restriction are greater than those raised by navigating with a mild restriction. In comparison with prior work, this suggests that the structure of the environment and complexity of paths affect spatial learning ability with impaired vision, with advantages seen in more regular and predictable environments. However, people with low vision must navigate through a variety of environment types that range in terms of structure, such as medical centers, entertainment venues, campus buildings, and open outdoor environments. Understanding the differential effects of environment type and the paths needed to traverse those environments on spatial learning ability with reduced visual information is important, to inform the design of potential navigational aids that could be used in varying settings.

## Data Availability

The datasets generated and/or analyzed during the current study are available in the Open Science Framework (OSF) repository (https://osf.io/5cena/).

## Abbreviations

FOV: Field of view
MEB: Merrill Engineering Building
RT: Reaction time
UMFA: Utah Museum of Fine Arts
